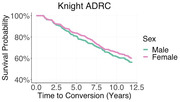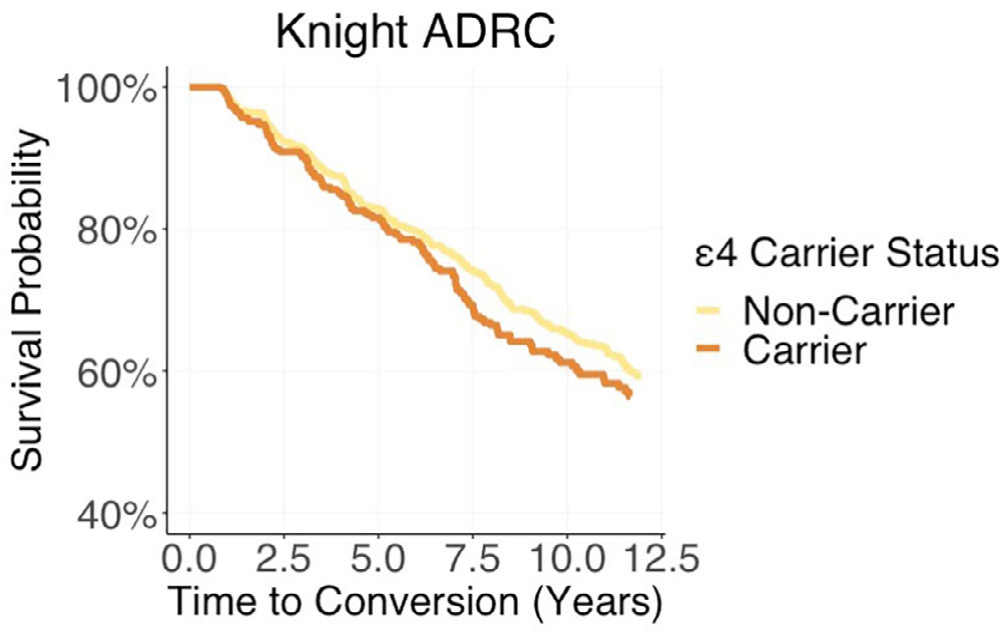# Sex differences in risk of developing Alzheimer Disease and cognitive outcomes

**DOI:** 10.1002/alz.093311

**Published:** 2025-01-09

**Authors:** Carling G. Robinson, John C. Morris, Jason J. Hassenstab, Carlos Cruchaga, Michael E. Belloy, Brian A. Gordon

**Affiliations:** ^1^ Washington University in St. Louis, St. Louis, MO USA; ^2^ Washington University School of Medicine in St. Louis, St. Louis, MO USA; ^3^ Washington University in St. Louis, School of Medicine, St. Louis, MO USA; ^4^ Department of Psychiatry, Washington University School of Medicine, St. Louis, MO USA; ^5^ Washington University in Saint Louis, Saint Louis, MO USA; ^6^ Washington University in St. Louis School of Medicine, St. Louis, MO USA

## Abstract

**Background:**

The prevalence of Alzheimer disease (AD) is growing rapidly within the aging population with over five million people currently affected in the United States. Of these 5 million people, approximately two‐thirds are females. Prior investigations have demonstrated that females have a higher likelihood of developing AD when compared to men. The present study sought to investigate participants from the Charles F. and Joanne Knight Alzheimer Disease Research Center (ADRC) to determine if sex impacts an individual’s risk of developing dementia.

**Method:**

3231 prospectively recruited participants from were classified as demented or non‐demented based on scores on the Clinical Dementia Rating ® (CDR®). Of the 3231 participants, 58.4 percent were female. Cox proportional hazards models were utilized to compare the rate of developing dementia as a function of self‐reported sex. Models additionally determine if *apolipoprotein ε4* (*APOE*) carriers were at a greater risk of developing dementia. We further evaluated the interaction between sex and *APOE* status and the likelihood of developing dementia. Baseline age was included as an additional covariate.

**Results:**

Participants had mean of 5.92 years of longitudinal cognitive follow‐up. Females did not demonstrate an increased risk of developing dementia compared to males (Hazard ratio = 0.874, p = 0.149). Age at baseline was a significant predictor of conversion to dementia (Hazard ratio = 1.100, p≤0.001), as was *ε4* carriers status (Hazard ratio = 1.64, p≤0.001). The interaction between female sex and *APOE* status was not a significant predictor of conversion to dementia (Hazard ratio = 1.034, p = 0.863).

**Conclusion:**

Female participants did not demonstrate an increased risk of developing all cause dementia compared to male participants.